# Information and Communications Technology–Based Monitoring Service for Tailored Chronic Disease Management in Primary Care: Cost-Effectiveness Analysis Based on ICT-CM Trial Results

**DOI:** 10.2196/51239

**Published:** 2024-10-11

**Authors:** Sung-Hee Oh, Jae-Heon Kang, Jin-Won Kwon

**Affiliations:** 1 Brain Korea 21 Four Community-Based Intelligent Novel Drug Discovery Education Unit, College of Pharmacy and Research Institute of Pharmaceutical Sciences Kyungpook National University Daegu Republic of Korea; 2 Department of Family Medicine, Kangbuk Samsung Hospital, College of Medicine Sungkyunkwan University Seoul Republic of Korea

**Keywords:** mHealth, mobile health, smartphone application, cost-effectiveness analysis, hypertension, diabetes mellitus, primary care, artificial intelligence, applications digital health, mobile phone

## Abstract

**Background:**

Information and communications technology–based tailored management (TM) intervention is a novel automatic system in which a smartphone app for the management of patients with hypertension and diabetes, the provider web, and Bluetooth devices are linked. However, little evidence exists regarding the cost-effectiveness of the interventions using mobile apps.

**Objective:**

This study aimed to assess the cost-effectiveness of TM intervention for adult patients with hypertension or diabetes in primary care compared with usual care (UC).

**Methods:**

Cost-effectiveness analysis using a Markov model was conducted from the Korean health care system perspective. Based on 6-month outcome data from an information and communications technology–based tailored chronic disease management (ICT-CM) trial, effectiveness over a lifetime beyond the trial periods was extrapolated using a cardiovascular disease risk prediction model. Costs were estimated using ICT-CM trial data and national health insurance claims data. Health utility weights were obtained from the Korea National Health and Nutrition Examination Survey.

**Results:**

In the base-case analysis, compared with UC, TM was more costly (US $23,157 for TM vs US $22,391 for UC) and more effective (12.006 quality-adjusted life-years [QALYs] for TM vs 11.868 QALYs for UC). The incremental cost-effectiveness ratio was US $5556 per QALY gained. Probabilistic sensitivity analysis showed that the probability of TM being cost-effective compared with UC was approximately 97% at an incremental cost-effectiveness ratio threshold of US $26,515 (KRW 35 million) per QALY gained.

**Conclusions:**

Compared with UC, TM intervention is a cost-effective option for patients with hypertension or diabetes in primary care settings. The study results can assist policy makers in making evidence-based decisions when implementing accessible chronic disease management services.

## Introduction

Hypertension and diabetes are major risk factors for cardiovascular diseases (CVD) associated with mortality and morbidity, imposing huge economic burdens [1,2]. Adequate blood pressure (BP) and glycemic control are important in health promotion and health care systems worldwide [3,4]. However, despite significant advances in evidence-based lifestyle modifications and pharmaceutical interventions, less than 40% of patients treated for hypertension or diabetes achieve the recommended target BP or blood glucose levels in Korea [5]. Therefore, novel interventions are needed to support patient efforts for behavior changes to promote healthy lifestyles and disease self-management [6,7]. Mobile health (mHealth) based on information and communication technology in primary care is an innovative approach to such interventions [8]. The widespread use of mobile phones combined with the ability to process and communicate data instantly enables real time individually tailored health care delivery and overcomes barriers of time and place [9].

A national pilot project for chronic disease management was recently conducted under the supervision of the Ministry of Health and Welfare to evaluate the effectiveness of information and communications technology (ICT)–based monitoring service for tailored chronic disease management in primary care in Korea [10,11]. The ICT-based tailored management (TM), using automated advanced systems for patients with hypertension and diabetes, provides a continuous and integrated customized health management service by linking the mHealth service platform (smartphone app) and the provider’s (primary care physician and care coordinator) operation web combined with the patient’s health information (examination and drug prescription information data from the National Health Insurance Service). Several mHealth interventions (eg, mobile phone SMS text messages, wearable monitoring devices, and telemedicine) for managing hypertension and diabetes have demonstrated efficacy [12], but evidence for interventions using smartphone apps is limited. Furthermore, their cost-effectiveness varied substantially based on the target disease and type of technology, and the results of economic evaluations have been inconsistent [13]. In particular, the cost-effectiveness of interventions using complex smartphone communications in older people cannot be judged due to a lack of information [14].

To our knowledge, no studies have examined the long-term cost-effectiveness of tailored management interventions for patients with hypertension or diabetes using automated advanced mobile technology in primary care. Although a large pilot project on TM intervention based on the highest smartphone penetration rate globally and advanced technology was conducted [15], it remains unclear whether the additional benefits from TM are sufficient to justify its use over and above usual care (UC) alone in terms of economic efficiency. This study aimed to estimate the cost-effectiveness of TM for adult patients older than 19 years of age with hypertension or diabetes in primary care compared with UC, using data collected in a pragmatic trial of ICT-based tailored chronic disease management (ICT-CM).

## Methods

### The ICT-Based Tailored Chronic Disease Management Trial

The ICT-CM was a pragmatic trial designed to test the real-world effectiveness of the ICT-based monitoring program among patients with chronic diseases in primary care [16]. Study participants were patients aged 19 years or older diagnosed with hypertension or diabetes, recruited from 8 clinics in Seoul and Gyeonggi-do, South Korea. Patients with myocardial infarction, stroke, end-stage renal disease, or liver failure within 1 year were excluded. Among the participants (n=1004) in the ICT-CM trial, those aged between 60 and <70 accounted for the highest proportion at 39% (392/1004), while those younger than 60 years accounted for 36.8% (369/1004), and those aged 70 and older accounted for 24.2% (243/1004). The average age was 62.4 years.

The study included a TM intervention group and a UC control group. Participants who received UC for hypertension and diabetes were treated at primary care institutions according to Korean clinical practice guidelines, and UC encompasses continuous monitoring and follow-up, appropriate medication management based on the patient’s condition, and lifestyle modifications to promote healthy habits [17,18]. Participants who received TM intervention were treated using automated advanced mHealth systems by physicians and care coordinators. A care coordinator trained participants who received TM on how to use the technology, downloaded the mobile application Carecrew (Huraypositive Inc) available for both Android (Google) and iOS (Apple Inc) onto their own smartphones, and were given Bluetooth-enabled devices (sphygmomanometer and glucometer). Physicians established personalized management goals for each patient (referred to as a tailored care plan) based on the lifestyle survey results and clinical examination at baseline. The care plan, including each participant’s target BP or blood glucose level, daily measurement frequency, and management priorities, was automatically sent to the Carecrew. The participants were asked to record their lifestyle such as diet and body weight and periodically check their blood glucose, BP, and medication use. Results measured by the devices were automatically uploaded to the provider’s operation web (CareCrew Web by Huraypositive Inc). The physicians and care coordinators had access to this system to monitor the patient’s condition constantly. Patients who missed self-recording were encouraged to self-measure and input lifestyle data through SMS text messages and phone calls. When the system reported out-of-range BP or glucose readings, a tailored mobile SMS text message was sent to the patient, and mobile-based feedback was performed for high-risk patients requiring intensive care. A customized examination voucher was issued for each patient and assessments were conducted at the clinic visit at 6 months. Source data from ICT-CM trial were verified through periodic on-site monitoring. Monitors identified any discrepancies between all case report form entries and the source data and issued queries. The queries were resolved by investigators, cooperating with monitors. The ICT-CM trial is described in detail in Multimedia Appendix 1.

### Ethical Considerations

The study was approved by the Investigation Review Board of Kangbuk Samsung Hospital (IRB KBSMC 2020-07-026-012). Informed consent and the ability of participants to withdraw were provided in the ICT-CM trial used for source data in this study. Expenses, including intervention costs and examination fees incurred due to study participation, were covered by the study, but no financial compensation was paid. All data used in this study were anonymized and deidentified.

### Model Overview

Long-term costs and health outcomes over the lifetime were compared for the TM intervention arm and UC arm. A target cohort population comprising Korean patients with hypertension or diabetes diagnosed in primary care, which was consistent with the populations examined in the ICT-CM trial. The Markov model was designed to mirror the natural progression of conditions in the population and the clinical pathway based on clinical guidelines. This model reflects the short-term outcomes of treatment (controlled-uncontrolled status of BP or blood glucose) in patients with hypertension or diabetes, their characteristics that were considered CVD risk factors, and the risk of long-term complications that patients may experience. The short-term outcomes were as observed in the ICT-CM trial at 6 months, and subsequent long-term outcomes were extrapolated from the trial data. The model was programmed in Microsoft Excel, supporting macro programming through Visual Basic for Applications. This study is reported according to the Consolidated Health Economic Evaluation Reporting Standards checklist [19].

### Model Structure

**Figure 1:**
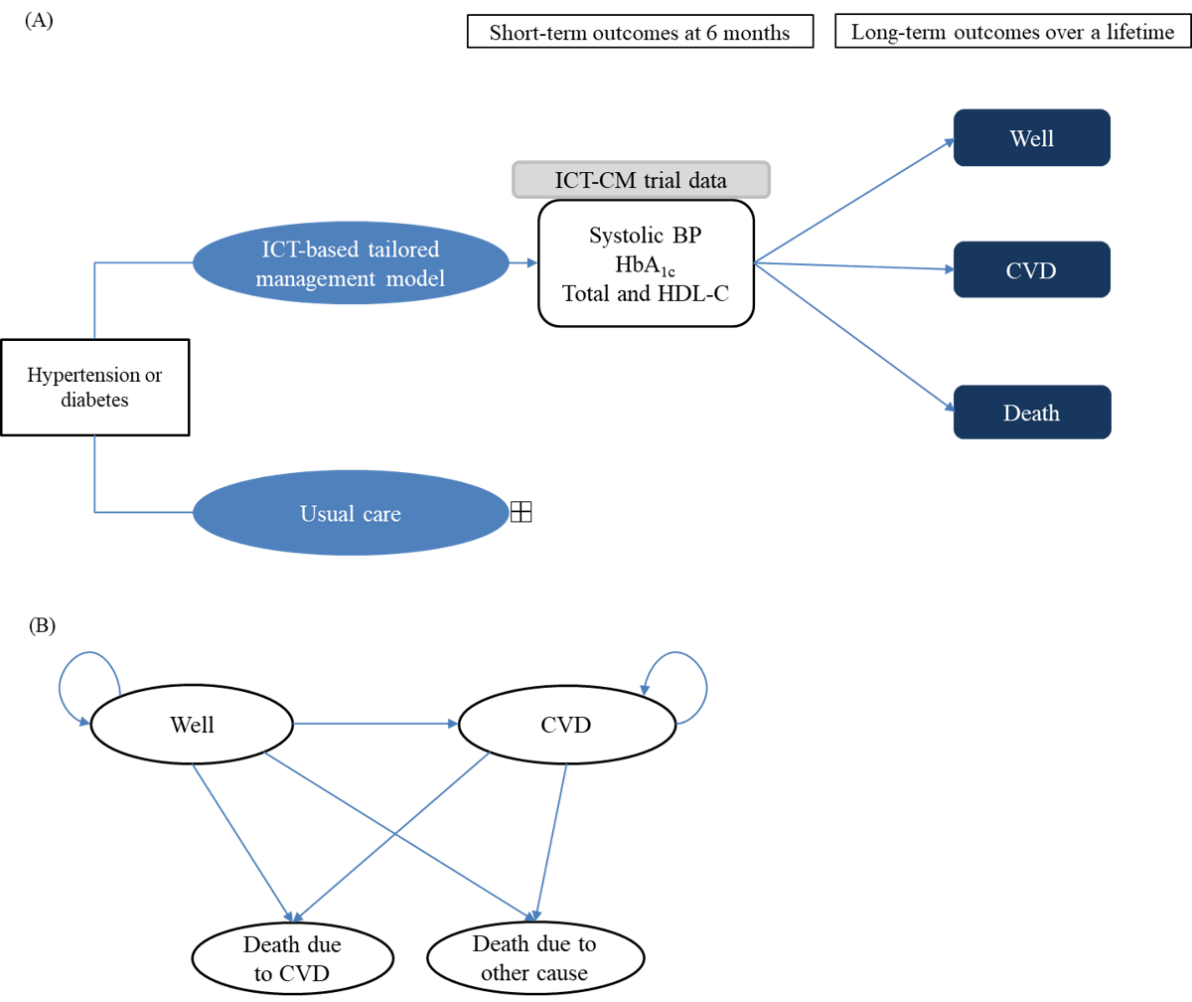
Model structure. (A) Model framework. The short-term outcomes were as observed in the ICT-CM trial at 6 months, with subsequent long-term CVD events with extrapolation from the trial data. (B) State transition diagram. The CVD was defined as a composite outcome of coronary heart disease (ie, myocardial infarction and angina), cerebrovascular diseases (ie, cerebral infarction, hemorrhagic stroke, and transient ischemic attack), heart failure, and peripheral artery disease. BP: blood pressure; CVD: cardiovascular diseases; HbA1c: glycated hemoglobin A1c; HDL-C: high-density lipoprotein cholesterol; ICT: information and communications technology; ICT-CM: information and communications technology–based monitoring service for tailored chronic disease management.

The structure of the Markov model is presented in Figure 1. The model structure’s validity was checked by reviewing the literature and expert opinion [20,21]. A Markov cohort could experience 1 of several health states, including “Well” with no major complications (ie, CVD), “CVD,” “Death due to CVD,” or “Death due to other cause.” “CVD” was defined as a composite outcome of coronary heart disease (ie, myocardial infarction and angina), cerebrovascular diseases (ie, cerebral infarction, hemorrhagic stroke, and transient ischemic attack), heart failure, and peripheral artery disease. All patients in the TM or UC groups started the Markov process in the “Well” state. Patients could remain in the “Well” state or transfer to another state at every cycle length of 6 months, corresponding to the frequency of assessments in the ICT-CM study. From any health state, patients could transit to the “Death due to other cause” state, and from the “CVD” state, patients could transit to the “Death due to CVD” state. Because the age of the patient cohorts on entry into the model was 60 years, a treatment discontinuation rate of 5% was assumed, considering that TM intervention could be discontinued in the real-world setting. Patients who discontinued TM intervention continued with UC. Patients receiving the UC at the model start were assumed to remain on the treatment until the end of the time horizon or death, whichever occurred first.

### Clinical Outcomes

The outcomes derived from the ICT-CM trial used in the study are presented in Table 1. Through post hoc analysis using individual patient data from the ICT-CM trial, the CVD risk factor outcomes were measured for each treatment arm. Demographic information including age and medical history were examined in screening tests, and smoking history was assessed in health surveys using a complete self-health questionnaire at both baseline and 6 months. Total cholesterol, high-density lipoprotein-cholesterol, and glycated hemoglobin A_1c_ (HbA_1c_) levels were assessed during clinical examinations at both baseline and 6 months, while BP was measured through body assessments at the same intervals. Details regarding the measurements and the outcomes assessed in the analysis are described in Multimedia Appendix 1. Using the input values associated with CVD risk factors obtained from the trial, the 10-year cardiovascular risk for each patient was calculated using the Framingham CVD risk prediction model [22]. The average risk estimate for each treatment arm was then converted into an annual probability of CVD events. The risk of CVD increased with the age of the patient cohort based on the age-related relative risk of CVD obtained from Kim et al [23]. The base-case regarding the long-term effect persistence assumed that the 6-month difference in BP or blood glucose between TM and UC decreased by 10% after 5 years, reflecting that the effectiveness may decline with age.

**Table 1:** Information and communications technology–based tailored chronic disease management trial participants’ data used in the Framingham risk model.

Risk factor outcomes	All patients with hypertension or diabetes			Patients with hypertension			Patients with diabetes	
	TM^a^	UC^b^	TM		UC	TM		UC
Male, %	52.8	50.5	49.5		46.1	57.0		54.9
Change from baseline to 6 months in systolic BP^c^, mean (mm Hg)	–1.6	4.3	4.9		3.3	2.7		5.4
Change from baseline to 6 months in diastolic BP, mean, mm Hg	–0.9	3.2	–2.9		3.9	1.6		2.3
Antihypertensive medication, %	55.6	55.5	100		99.4	66.4		63.2
Change from baseline to 6 months in total cholesterol, mean (mg/dL)	–2.4	–1.1	–5.7		–1.5	1.8		–0.7
Change from baseline to 6 months in HDL^d^-cholesterol, mean (mg/dL)	1.3	1.8	1.1		1.2	1.6		2.4
Change from baseline to 6 months in HbA_1c_^e^, mean (%)	–0.1	–0.1	–0.1		–0.0	0.0		–0.1
Current smoker, %	18.5	13.7	15.0		12.9	23.2		17.9

^a^TM: information and communications technology–based tailored management.

^b^UC: usual care.

^c^BP: blood pressure.

^d^HDL: high-density lipoprotein.

^e^HbA_1c_: glycated hemoglobin A_1c_.

All-cause and CVD-related mortality of the general population were based on cause-of-death statistics by age of the Korean Statistical Office [24]. The annual probability of death due to other-cause was calculated by excluding death due to CVD from all-cause death. The fatality rate for CVD by age was estimated as the number of deaths due to CVD among patients with CVD, using the cause-of-death statistics of the Korean Statistical Office [24] and the Korean National Health Insurance statistics by the Health Insurance Review and Assessment Service [25]. Table 2 presents a detailed description of the model inputs.

**Table 2:** Model inputs for patients with hypertension or diabetes.

Parameters					Estimate in base-case		Distribution^a^		Sources
**Analysis setting**									
	Start age (year)				60		—^b^		ICT-CM^c^ trial population
	Time horizon (year)				Lifetime		—		Assumed
**Mortality and risk of CVD^d^**									
	**Probability of death due to CVD, %**								Cause-of-death statistics of Korean Statistical Office; Korean national health insurance statistics by HIRA^e^
		60-69 years		0.72		Beta			
		70-79 years		1.36		Beta			
		≥80 years		5.72		Beta			
	**Probability of death due to other cause, %**								Cause-of-death statistics of Korean Statistical Office
		60-69 years		0.61		Beta			
		70-79 years		1.65		Beta			
		≥80 years		7.01		Beta			
	**Probability of CVD events (1-year risk)^f^, %**								ICT-CM trial and Framingham CVD risk prediction model
		**TM^g^**							
			60-64 years	0.90		Beta			
			65-69 years	0.90		Beta			
			70-74 years	1.32		Beta			
			75-79 years	1.32		Beta			
			≥80 years	1.80		Beta			
		**Usual care**							
			60-64 years	1.37		Beta			
			65-69 years	1.37		Beta			
			70-74 years	1.91		Beta			
			75-79 years	1.91		Beta			
			≥80 years	2.59		Beta			
	**Age-related relative risk of CVD**								Published data using national health insurance claims data by HIRA (Kim et al [23])
		60-64 years		1.00		—			
		65-69 years		1.39		—			
		70-74 years		1.90		—			
		75-79 years		2.17		—			
		≥80 years		2.06		—			
**Utility weights, mean (SE)**									2007-2019 KNHANES^h^ database
	**CVD**								
		60-69 years		0.859 (0.006)		Beta			
		70-79 years		0.794 (0.008)		Beta			
		≥80 years		0.747 (0.016)		Beta			
	**Well**								
		60-69 years		0.914 (0.002)		Beta			
		70-79 years		0.858 (0.003)		Beta			
		≥80 years		0.802 (0.006)		Beta			
**Intervention costs (US $^i^)**									ICT-CM trial
	**Bluetooth-enabled devices costs per patient, one-off**								
		Sphygmomanometer		54.5		Gamma			
		Glucometer		34.1		Gamma			
	**Equipment costs**								
		Initial development costs of application and operation web per 5 years		757,575.8		Gamma			
		Annual network use costs		227,272.7		Gamma			
		Annual system update costs		145,454.5		Gamma			
		Total costs per patient, annuitized based on lifespan of 5 years		2.3		—			
	Running costs per patient^j^				141.8		Gamma		
**Treatment costs of health states (US $)**									Analysis using national health insurance claims data by NHIS^k^
	**Acute phase CVD (first year costs after event)**								
		60-64 years		2676.7		Gamma			
		≥65 years		3639.8		Gamma			
	**Chronic phase CVD (annual costs in subsequent years)**								
		60-64 years		1524.0		Gamma			
		≥65 years		2580.1		Gamma			
	**Well (annual costs)**								
		60-64 years		960.6		Gamma			
		≥65 years		1566.9		Gamma			

^a^Distributions used in probabilistic sensitivity analysis.

^b^Not applicable.

^c^ICT-CM: information and communications technology–based tailored chronic disease management.

^d^CVD: cardiovascular diseases.

^e^HIRA: Health Insurance Review and Assessment Service.

^f^In base-case for all patients, the 10-year cardiovascular risk was calculated with Framingham CVD risk prediction models based on the CVD risk factors obtained from the ICT-CM trial. 1-year probability was calculated from the 10-year risk values.

^g^TM: information and communications technology–based tailored management.

^h^KNHANES: Korea National Health and Nutrition Examination Survey.

^i^All costs are expressed in 2023 US $ using an exchange rate of US $1=1320 KRW.

^j^Through the TM trial, the annual running cost was calculated by dividing the care coordinator’s labor cost by the number of managed patients (approximately 250 per year).

^k^NHIS: National Health Insurance Service.

### Utility Weights

The utility weights for the health states of patients with hypertension or diabetes were derived from the Korea National Health and Nutrition Examination Survey (KNHANES, 2007-2019) data conducted by the Korea Centers for Disease Control and Prevention. KNHANES is a nationwide survey conducted annually for the representative general population in Korea [26]. The survey includes individual-level information on health-related quality of life measured by the EQ-5D developed by the EuroQol Group. The 5 dimensions of EQ-5D-3L comprise mobility, self-care, usual activities, pain or discomfort, and anxiety or depression, and each dimension was answered with 3 levels. The health state index scores calculated based on the tariff of Lee et al [27] range from 0 (where 0 is a health state equivalent to death) to 1 (perfect health), with higher scores indicating higher health utility. The utility weight of “Well” status was higher than that of “CVD” by 0.05 for patients in their sixties, and the utility weight decreased with age. However, the difference in utility weight between the 2 health states remained similar. The utility weights by age are presented in Table 2.

### Costs

All costs were expressed in 2023 US $ using an exchange rate of US $1=1320 KRW and adjusted for inflation where applicable using the Consumer Price Index for health care [28]. Intervention costs of patients who received TM were obtained from the TM trial. Bluetooth-enabled device costs per patient were applied as one-off costs to the model’s initial health state (“Well”). Equipment costs included initial development, network use, and system update expenditure. The per capita total equipment costs were applied to the model, estimated based on the patient number of target cohorts reflecting smartphone penetration [23]. Running costs comprised costs associated with training and care coordinator. Through the TM trial, the number of manageable patients per care coordinator was investigated, and the annual running cost was calculated by dividing the care coordinator’s labor cost by the number of managed patients (approximately 250 per year). Running and equipment, annuitized based on a lifespan of 5 years, were included in intervention costs over the lifetime time horizon.

The cost of “Well” and “CVD” health states were derived from data analysis of the national health insurance claims data [29], and were applied equally to the 2 arms. Each health state’s costs comprised expenditure for diagnostic test, medication, surgical treatments, and resources use associated with inpatient or outpatient services. The costs of cardiovascular events including initial acute care and long-term care were estimated. The costs of acute phase CVD were applied to the model in the first year after event, and in subsequent years, annual costs of chronic phase CVD were applied. All cost inputs are presented in Table 2.

### Cost-Effectiveness Analysis

In a base-case analysis of all patients with hypertension or diabetes, the cost-effectiveness analysis of TM compared with UC was conducted from the health care system perspective. A result was presented as an incremental cost-effectiveness ratio (ICER) calculated by dividing the incremental cost by the additional quality-adjusted life-year (QALY) gained. A 4.5% annual discount rate for both cost and QALY were performed with half-cycle corrections. Deterministic and probabilistic sensitivity analysis (PSA) was performed to address parameter uncertainty. The parameters, including probabilities, utility weights, costs, number of manageable patients per care coordinator, and discount rate, were varied through deterministic sensitivity analyses. PSA of 1000 Monte Carlo simulations was undertaken, in which each of the parameter estimates was sampled from its distribution listed in Table 2. PSA results were expressed as a cost-effectiveness plane and cost-effectiveness acceptability curve that shows graphically the probability of cost-effectiveness for all alternatives across a range of an ICER threshold of US $26,515 (KRW 35 million) per QALY [30]. In addition, several scenarios were explored to assess the structural uncertainty associated with model assumptions. The assumptions regarding the effect persistence between the 2 arms were tested by varying the period during which the 6-month effect difference lasts, from the 3 years beyond the trial period to the remainder of the lifetime. In consideration of treatment persistence, the scenario considering a treatment discontinuation rate of 0% or 10% in patients receiving TM was also evaluated. Finally, the model’s time horizon varied from a lifetime to between 10 and 20 years.

A subgroup analysis was performed for patients with hypertension and diabetes. The Framingham risk model used in the base-case was reported to overestimate the risk of CVD in Koreans [31], and underestimate the risk of CVD in patients with diabetes [32]. Therefore, other CVD risk models were used in each subgroup. The Korean Hypertension cardiovascular (KH-CVD) risk model for patients with hypertension [33] and the United Kingdom prospective diabetes study (UKPDS) model that estimates the risk of coronary heart disease and stroke [32,34] for patients with diabetes were used to predict long-term effects. Table S1 in Multimedia Appendix 2 shows model inputs, such as probability of CVD events, utility, and costs, estimated for each subgroup.

## Results

### Base-Case Analysis

Compared with UC, the cost-effectiveness analysis indicated that TM was more effective (12.006 QALYs for TM vs 11.868 QALYs for UC) and more costly (US $23,157.4 for TM vs US $22,390.5 for UC). TM had incremental costs of US $766.9 and incremental QALYs of 0.138 compared with UC over the lifetime horizon for patients with hypertension or diabetes in South Korea. Through the base-case analysis, ICER was US $5556 per QALY gained (Table 3).

**Table 3:** Cost-effectiveness results in base-case and sensitivity analyses.

						Cost (US $^a^)			Incremental cost (US $)			QALYs^b^			Incremental QALYs			ICER^c^ (US $/QALY gained)
**Base-case analysis**																		
	UC^d^				22,390.5			—^e^			11.868			—			—	
	TM^f^				23,157.4			766.9			12.006			0.138			5556	
**Sensitivity analyses**																		
	**Probability of CVD^g^ events**																	
		**Increase by 20% for TM**																
			UC	22,390.5			—			11.868			—			—		
			TM	23,467.8			1077.3			11.950			0.082			13,167		
		**Decrease by 20% for TM**																
			UC	22,390.5			—			11.868			—			—		
			TM	22,824.5			433.9			12.067			0.198			2188		
		**Increase by 20% for UC**																
			UC	22,808.9			—			11.794			—			—		
			TM	23,190.3			381.4			12.000			0.207			1846		
		**Decrease by 20% for UC**																
			UC	21,923.1			—			11.952			—			—		
			TM	23,124.2			1201.1			12.012			0.061			19,787		
	**Utility weights**																	
		**Increase by 20%**																
			UC	22,390.5			—			13.454			—			—		
			TM	23,157.4			766.9			13.584			0.130			5901		
		**Decrease by 20%**																
			UC	22,390.5			—			9.495			—			—		
			TM	23,157.4			766.9			9.605			0.110			6945		
	**Costs**																	
		**Increase by 20% for TM**																
			UC	22,390.5			—			11.868			—			—		
			TM	23,558.5			1167.9			12.006			0.138			8462		
		**Decrease by 20% for TM**																
			UC	22,390.5			—			11.868			—			—		
			TM	22,756.4			365.8			12.006			0.138			2651		
	**Number of manageable patients per care coordinator**																	
		**Increase by 20%**																
			UC	22,390.5			—			11.868			—			—		
			TM	22,833.0			442.5			12.006			0.138			3206		
		**Increase by 40%**																
			UC	22,390.5			—			11.868			—			—		
			TM	22,611.0			220.4			12.006			0.138			1597		
	**Discount rate**																	
		**0%**																
			UC	37,710.2			—			18.342			—			—		
			TM	38,777.1			1066.9			18.637			0.295			3612		
		**3%**																
			UC	26,311.4			—			13.555			—			—		
			TM	27,155.7			844.3			13.731			0.176			4803		
	**Effect persistence for TM**																	
		**Lasts 3 years beyond the trial period**																
			UC	22,390.5			—			11.868			—			—		
			TM	23,672.9			1282.3			11.914			0.046			28,170		
		**Lasts 4 years beyond the trial period**																
			UC	22,390.5			—			11.868			—			—		
			TM	23,625.5			1235.0			11.922			0.053			23,192		
		**Lasts 5 years beyond the trial period**																
			UC	22,390.5			—			11.868			—			—		
			TM	23,566.1			1175.6			11.931			0.063			18,631		
		**Decrease by 10% per year beyond the trial period**																
			UC	22,390.5			—			11.868			—			—		
			TM	23,183.3			792.7			12.002			0.133			5940		
		**Decrease by 10% per year after 3 years**																
			UC	22,390.5			—			11.868			—			—		
			TM	23,168.2			777.6			12.005			0.136			5708		
		**Decrease by 10% per year after 7 years**																
			UC	22,390.5			—			11.868			—			—		
			TM	23,145.1			754.6			12.008			0.140			5386		
		**Last over a lifetime**																
			UC	22,390.5			—			11.868			—			—		
			TM	23,100.5			710.0			12.017			0.148			4786		
	**Treatment persistence**																	
		**10% discontinuation rate for TM**																
			UC	22,390.5			—			11.868			—			—		
			TM	23,180.2			789.6			12.006			0.138			5721		
		**0% discontinuation rate for TM**																
			UC	22,390.5			—			11.868			—			—		
			TM	23,154.4			763.9			12.006			0.138			5535		
	**Time horizon**																	
		**10 years**																
			UC	11,081.9			—			7.326			—			—		
			TM	11,696.7			614.7			7.350			0.024			25,201		
		**20 years**																
			UC	19,825			—			10.960			—			—		
			TM	20,519.2			694.3			11.050			0.090			7731		

^a^All costs are expressed in 2023 US $ using an exchange rate of US $1=1320 KRW.

^b^QALYs: quality-adjusted life-years.

^c^ICER: incremental cost-effectiveness ratio.

^d^UC: usual care.

^e^Not applicable.

^f^TM: information and communications technology–based tailored management.

^g^CVD: cardiovascular diseases.

### Sensitivity Analysis

In the results of the sensitivity analyses, the ICER was mainly affected by the effect persistence of TM, time horizon, the probability of CVD events for UC, the probability of CVD events for TM, and TM intervention costs, in that order (Table 3 and Figure S1 in Multimedia Appendix 2). When a 10% decrease in the TM intervention’s effectiveness was applied at 6 months and 7 years after the start of the intervention, all ICERs remained below a threshold of US $26,515. However, when the additional effect due to TM intervention disappeared at 3 to 5 years beyond the trial period, the ICER increased above the threshold after 3 years. The ICER was the highest at US $25,201/QALY in the 10-year time horizon scenario. When the probability of CVD events for UC was decreased by 20%, the ICER increased by 256.1% (US $19,787/QALY). As the time when horizon and the probability of CVD events for UC decreased, the additional QALY gains in TM decreased significantly. A 20% decrease in the TM intervention costs and an increase in manageable patients per care coordinator showed cost-saving results. However, TM was the cost-effective strategy in all variations assessed in the sensitivity analyses, except for the assumption that the TM effect lasts only 3 years.

**Figure 2:**
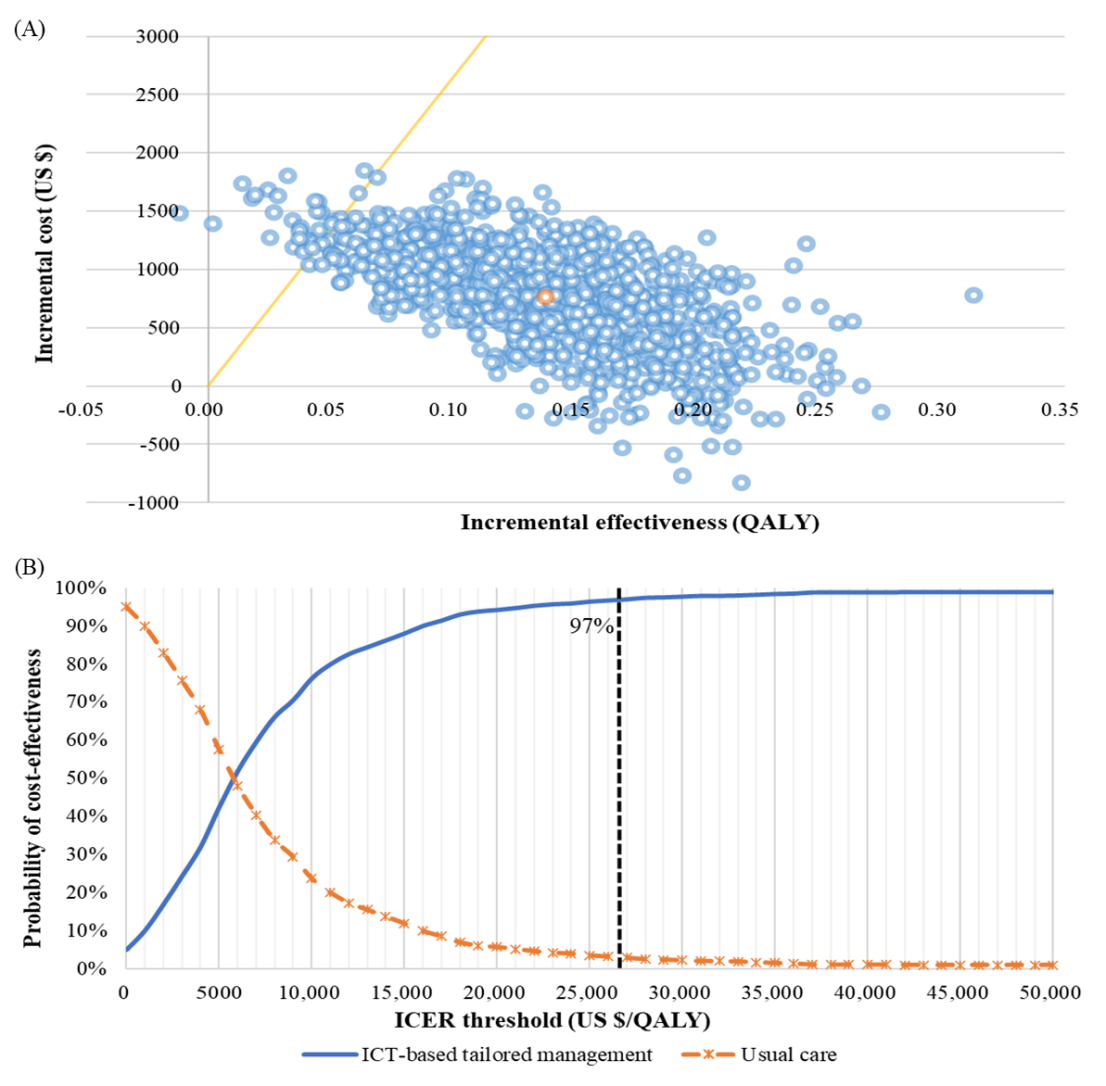
Results of probabilistic sensitivity analysis. (A) Cost-effectiveness plane. (B) Cost-effectiveness acceptability curve. Dotted line on graph indicates an incremental cost-effectiveness ratio threshold. ICER: incremental cost-effectiveness ratio; ICT: information and communications technology; QALY: quality-adjusted life-year.

The results of the PSA were presented in cost-effectiveness planes showing the distributions of incremental costs and effects between the 2 arms (Figure 2A). Most values were distributed below the cost-effective threshold in the northeast quadrant, indicating that the base-case results were robust. The cost-effectiveness acceptability curve (Figure 2B) showed that the probability of the ICER being below the threshold of US $26,515 per QALY gained was 97%.

### Subgroup Analysis

In the subgroup analysis of patients with hypertension, TM had US $678.7 higher costs and 0.154 higher QALYs than UC, presenting an ICER gain of US $4417 per QALY. In patients with diabetes, TM had US $885 higher costs and 0.102 higher QALYs than UC, presenting an ICER gain of $8687 per QALY (Table 4). The probability that TM would be cost-effective was 99% for treating patients with hypertension, and 86% for patients with diabetes (Figure S2 in Multimedia Appendix 2). When the long-term effect was estimated using the KH-CVD model for patients with hypertension and the UKPDS model for patients with diabetes, the ICER gain was US $8699 per QALY for patients with hypertension and US $10,409 per QALY for patients with diabetes. Consequently, TM was a cost-effective alternative in all subgroups (Table 4).

**Table 4:** Cost-effectiveness results for subgroups of patients with hypertension and diabetes.

			Cost (US $^a^)	Incremental Cost (US $)	QALYs^b^	Incremental QALYs	ICER^c^ (US $/QALY gained)
**Patients with** **hypertension**							
	**Long-term effect estimated using the Framingham model**						
		UC^d^	22,502	—^e^	11.848	—	—
		TM^f^	23,180.6	678.7	12.002	0.154	4417
	**Long-term effect estimated using the KH-CVD^g^ model**						
		UC	20,710.9	—	12.024	—	—
		TM	21,637.1	926.2	12.131	0.106	8699
**Patients with** **diabetes**							
	**Long-term effect estimated using the Framingham model**						
		UC	26,537.5	—	11.789	—	—
		TM	27,422.7	885.2	11.891	0.102	8687
	**Long-term effect estimated using the UKPDS^h^ model**						
		UC	25,746.1	—	11.913	—	—
		TM	26,703.2	957.1	12.005	0.092	10,409

^a^All costs are expressed in 2023 US $ using an exchange rate of US $1=1320 KRW.

^b^QALYs: quality-adjusted life-years.

^c^ICER: Incremental Cost Effectiveness Ratio.

^d^UC: usual care.

^e^Not applicable.

^f^TM: information and communications technology–based tailored management.

^g^KH-CVD: Korean Hypertension cardiovascular.

^h^UKPDS: United Kingdom prospective diabetes study.

## Discussion

### Principal Findings

In the economic evaluation model based on the CVD Risk Prediction Model with data from the ICT-CM pragmatic trial, we assessed the cost-effectiveness of TM for patients with hypertension or diabetes in primary care, compared with UC. The study found that TM, despite being more costly than UC, resulted in a better quality of life due to reduced cardiovascular events, making it a cost-effective option. PSA indicated that the probability of the ICER value lying below the threshold WTP was 97%.

Mobile technology is increasingly used to manage the chronic diseases of hypertension and diabetes because of its efficacy and accessibility [35]. Several devices using mobile technology improve patient care and health outcomes through lifestyle modification, promoting behavioral changes and increasing medication adherence [12]. Previous studies have shown that mHealth interventions using mobile phone SMS text messages, wearable monitoring devices, and telemedicine are cost-effective in managing chronic diseases [13]. With the continuous advance of technology, novel mobile systems using software programs such as smartphone apps and devices have recently emerged in Japan [36], and these have proven to be cost-effective for reducing BP [37]. Our study confirmed that automated algorithm-based advanced systems, in which mobile app for patients with hypertension and diabetes, patient management software for primary doctors, and Bluetooth-enabled devices were linked, had better treatment outcomes. This study is significant in that it proved the cost-effectiveness of the automated advanced mHealth system in patients with hypertension and diabetes in South Korea.

The model extrapolated effects over a lifetime based on the intervention effects at 6 months after starting the trial. The initial improvement in the HbA_1c_ levels at 6 months in the tailored mobile coaching group, including a mobile app, continued until 12 months in the randomized controlled trial despite partial cessation of interventions [38]. Another study showed that tailored phone- and home-monitoring interventions resulted in a continued greater improvement in systolic BP compared with usual care over a 2-year period [39]. Therefore, it was assumed in a base-case to be a 10% decrease in effect difference between the TM and UC groups after 5 years from the start of the intervention, which seems reasonable. In the sensitivity analyses that explored the assumption of effect persistence, the study demonstrated that the TM intervention in primary care was cost-effective compared with UC, even though the effect difference decreased by 10% immediately after the trial period. Conversely, if the added benefits of the TM intervention over UC were eliminated after 3 years, the intervention would not be cost-effective. However, as the discontinuation rate of TM intervention was expected to be low (conservatively, 5% was applied in the model), the assumption that the effect disappears after 3 years despite ongoing treatment is considered unlikely in the real world.

In the subgroup analysis, patients with diabetes were associated with higher incremental costs and lower incremental QALYs in the TM group versus the UC group compared with patients with hypertension. Reduced equipment costs per capita due to a higher prevalence of hypertension than diabetes [23] may have contributed slightly to this result. Furthermore, 6-month outcomes, such as systolic BP and total cholesterol of the TM group compared with the UC group, improved more in patients with hypertension than in patients with diabetes, which would have resulted in better long-term effects and better QALY in patients with hypertension. While the rate of reduction in BP is relatively rapid after treatment [40], significant changes in HbA_1c_ may not occur in the short-term period after treatment change as A_1c_ reflects average glycemia over several months [41]. In a study evaluating long-term changes in HbA_1c_ for 7 years after visiting a tertiary hospital in patients with diabetes, HbA_1c_ decreased most strongly at 12 months and then was maintained thereafter [42]. Thus, in the subgroup analysis for patients with diabetes, the effect of the TM group might be underestimated compared with UC group. Nevertheless, our study found an 86% probability that the TM group would be cost-effective among patients with diabetes. Hypertension and diabetes are common comorbidities; each accelerates the other by being linked through vascular mechanisms and risk factors [43]. Therefore, implementing the TM program for patients with chronic illnesses such as hypertension or diabetes would efficiently prevent and manage CVD.

The Framingham model was used to estimate the long-term CVD outcome in a base-case, which is widely used to predict the risk of developing CVD worldwide. However, it has been reported to overestimate the CVD risk in the Asian population [31,44]. The accuracy of the Framingham model for patients with diabetes has also been debated [32]. As only 6% of the Framingham cohort was known to have diabetes, it was reported that the Framingham model tended to underestimate the risk in patients with diabetes [45]. Therefore, the study used other CVD risk models to evaluate the uncertainty due to the risk model. The KH-CVD model for patients with hypertension was developed based on the data from Korean Hypertension Cohort of 11,043 patients who were followed for 10 years at 6 national university hospitals, and the model validation demonstrated moderate predictive accuracy [33]. In tertiary hospitals, the risk of CVD may be underestimated due to strict patient management. The UKPDS model includes HbA_1c_ as a continuous variable, while the Framingham model includes dichotomous variables such as the presence or absence of diabetes as risk factors. The UKPDS model, which is sensitive to HbA_1c_ improvement, demonstrated enhanced precision in predicting CVD among patients with diabetes [32]. It exhibited acceptable discriminative performance for CVD [46]. However, the 6-month outcome from the ICT-CM trial in our study was insufficient to confirm significant improvement in HbA_1c_; thus, it is speculated that the difference in effect between the 2 groups was underestimated. Hence, the ICER increased when estimating the CVD risk using the KH-CVD and UKPDS models compared with using the Framingham model. However, our results indicated that there was no significant change in the cost-effectiveness of TM.

The intervention cost of mHealth is a crucial factor for cost-effectiveness. Nomura et al [37] indicated that the cost of mobile app–based intervention for hypertension had the greatest impact on the ICER [37]. Although our deterministic sensitivity analysis results showed that the cost of TM intervention was not the most determinant, similar to previous studies [37,47], a reduction in intervention costs resulted in good cost-effectiveness. In particular, it might be possible that annual running costs, which account for the largest share of the TM intervention costs, could substantially decrease over time. The number of patients managed per care coordinator is expected to increase as the automation model using artificial intelligence is enhanced and care coordinators become more proficient, enabling efficient operation. This leads to reduced running costs and greater cost-effectiveness of intervention. The study found that the ICER decreased by more than 70% when the number of managed patients increased by 40% from 250 to 350 per year.

### Limitations

Our study has some limitations. First, the TM intervention effect after the observed periods of the ICT-CM trial was unknown, but it was assumed to last for 5 years and then decline in the base-case. Our sensitivity analyses showed that the results remained robust to various potential reductions in effect, with the exception of no additional effect of TM after 3 years, which might be perceived as a very conservative approach. Further studies are needed to support the long-term use of various mHealth systems. Second, we used a Framingham CVD risk prediction model that was not specifically designed to predict outcomes among adults in South Korea. However, sensitivity analysis using the KH-CVD model developed in Korea demonstrated that TM intervention would still be cost-effective. Furthermore, the Framingham model has been retrospectively validated in a multiethnic Asian population in primary care settings in a 10-year cohort study [48]. Third, we could not consider whether cost-effectiveness differs in relation to lifestyle and metabolic risk changes by the amount of app usage. Nevertheless, various sensitivity analyses supported that the base-case result was robust.

### Conclusions

From a Korean health care system perspective, TM intervention is a cost-effective strategy compared with UC in patients with hypertension or diabetes in primary care settings. The study can help policy makers seeking to implement accessible chronic disease management services reach at evidence-based decision-making.

## Appendix

Multimedia Appendix 1 Information and Communications Technology–based tailored chronic disease management (ICT-CM) trial.

Multimedia Appendix 2 Model inputs for subgroups of patients with hypertension and diabetes. Tornado diagram for TM versus UC. Results of probabilistic sensitivity analysis for subgroups of patients with hypertension and diabetes. (A) Cost-effectiveness plane for patients with hypertension. (B) Cost-effectiveness acceptability curve for patients with hypertension. (C) Cost-effectiveness plane for patients with diabetes. (D) Cost-effectiveness acceptability curve for patients with diabetes.

## Abbreviations

BP: blood pressure
CVD: cardiovascular diseases
HbA1c: glycated hemoglobin A1c
ICER: incremental cost-effectiveness ratio
ICT-CM: information and communications technology–based tailored chronic disease management
TM: information and communications technology–based tailored management
KH-CVD: Korean Hypertension cardiovascular
KNHANES: Korea National Health and Nutrition Examination Survey
mHealth: mobile health
PSA: probabilistic sensitivity analysis
QALY: quality-adjusted life-year
UC: usual care
UKPDS: United Kingdom prospective diabetes study

## Data Availability

The datasets generated and analyzed during this study are available from the corresponding author upon reasonable request.
