# Supplementary material for: Information and Communications Technology–Based Monitoring Service for Tailored Chronic Disease Management in Primary Care: Cost-Effectiveness Analysis Based on ICT-CM Trial Results
Source: J Med Internet Res. 2024 Oct 11;26:e51239. doi: 10.2196/51239 (PMC11512140; doi:10.2196/51239)
Supplement: Multimedia Appendix 1 [file jmir_v26i1e51239_app1.docx]

**ICT-based tailored chronic disease management (ICT-CM) trial**

-A national pilot project on ICT-based tailored management intervention based on advanced IT technology-

**1. Study objectives**

Evaluating the effectiveness of an ICT-based monitoring program among patients with chronic diseases in primary care

**2. Investigator**

-Name: Jae-Heon Kang, MD, PhD

-Address: Department of Family Medicine, Kangbuk Samsung Hospital, College of Medicine, Sungkyunkwan University

29, Saemunan-ro, Jongno-gu, Seoul, Republic of Korea

**3. Duration of study**

-Duration of study: 2021.01.08 ~ 2024.01.07

**4. Study centers:** 8 sites in Seoul and Gyeonggi-do, South Korea

**5. Study design and plan**

-Patients diagnosed with diabetes or hypertension at 8 clinics in Seoul and Gyeonggi-do, South Korea between May 2 and August 5, 2022, were recruited.

-During duration of patient participation of 6 months (2022.06.03~23.02.01), the 3 visits took place in the intervention group. Assessments were completed according to the following schedule:

Visit 1 at baseline: consent form, health survey, pre-examination

Visit 2 at 12 weeks: Interim examination

Visit 3 at 24 weeks: final examination

-In the control group, patients visited at clinic for routine care during the participation period, and no additional visits were required.

**6. Study population (enrollment criteria)**

○Inclusion Criteria

A patient who met all of the following criteria qualified for entry into the study:

1) 19 years and older.

2) Diagnosis with hypertension or type 2 diabetes in primary care institutions.

3) Patients who understand the purpose of the study and are capable of reading and writing.

4) Patients capable of using the smartphone application used in the study.

5) Voluntary participant and patients who have signed the informed consent form.

○Exclusion Criteria

A patient who met any of the following criteria did not qualify for entry into this study:

1) Patients participating in the hypertension and diabetes registration management project.

2) Patients hospitalized or expected to require hospitalization within the study period.

3) Diagnosed with myocardial infarction or stroke within the last year.

4) Patients with end-stage renal failure requiring renal replacement therapy, with serum creatinine levels exceeding 1.5 times the upper limit.

5) Patients with liver failure (severe liver function impairment patients), with serum liver enzyme levels (AST or ALT) exceeding 5 times the upper limit.

6) Patients with uncontrolled chronic pulmonary disease.

7) Patients taking medications (psychiatric drugs, etc.) that may affect weight.

8) Patients with any significant morbidity or condition which may affect study results or interfere with the study participation.

**7. Intervention arm/control arm**

**(1) ICT-based tailored management (TM) intervention group**

-TM intervention, using an automated advanced system, provides customized health management services by linking with a mobile health service platform (smartphone application CareCrew by Huraypositive Inc) and the provider web. The provider web, designed for primary physicians and care coordinators, was integrated with the patient's health information, including national examination and prescription data. Tailored care plans for each patient were established by the physician; the CareCrew app automatically provided personalized health messages, such as pop-up messages for lifestyle improvement and missing record notifications. The physician and care coordinator continuously monitor the patient's condition and manage the patient periodically (monthly for the physician, weekly for the care coordinator). Motivational and risk management messages were sent to patients, and telephone consultations were provided when necessary.

**Smartphone app CareCrew by Huraypositive Inc**

-A mobile application that enables physicians and care coordinators to monitor and educate based on self-measured blood pressure, blood glucose, meals, exercise, and health examination data.

○ Data collected by the app include:

1) Health indicators: blood pressure, blood glucose, weight (linked to device), waist circumference/blood test results (linked to web).

2) Meal records: types of food, calorie intake, photo input.

3) Activity data: step count.

4) Exercise information: type of exercise, calories burned/ingested.

5) Medication information.

6) Other indicators such as smoking, drinking, etc.

○ Services provided by the app include:

1) Calorie intake based on dietary input.

2) Health information for chronic disease management.

3) Statistics based on lifestyle recorded for one week (weight, blood glucose, blood pressure, meals, exercise, step count).

4) Online coaching: providing personalized health messages based on the input life log through the app.

5) Response from specialized medical staff regarding health-related inquiries.

**(2) Usual care (UC) control group**

-Eligible participants receiving routine care at primary care institution.

-Treated according to Korean clinical practice guidelines.

**8. Measurements**

**(1)** **Screening test**

-Demographic information including gender, age, smoking, alcohol consumption.

-Medical history within 1 year prior to screening visit or comorbidities.

-Medication history within 4 weeks prior to screening visit, including name, daily dose, treatment duration, indication.

**(2)** **Health survey**

-Complete self-health questionnaire at baseline and 6 months.

-Survey items: Dietary, lifestyle (smoking, exercise, etc.).

-Dietary evaluation: Comparison of pre and post-dietary surveys and meals recorded in the application.

-Lifestyle evaluation: Comparison of pre and post-lifestyle surveys and exercise recorded in the application.

**(3) Clinical examination**

-Assessments at baseline and 6 months.

Examination items include:

-Complete blood count: Red Blood Cells (RBC), White Blood Cells (WBC), Platelets, glycated hemoglobin A1c (HbA1c), Hematocrit (Hct), Mean Corpuscular Hemoglobin (MCH), Mean Corpuscular Volume (MCV), Mean Corpuscular Hemoglobin Concentration (MCHC), Red Cell Distribution Width (RDW).

-Blood Chemistry: Glucose (fasting), TG (Triglyceride), T-Cholesterol, HDL-Cholesterol, LDL-Cholesterol, BUN (Blood Urea Nitrogen), Creatinine, GOT(AST), GPT(ALT)

-Urinalysis: Color, S.G (Specific Gravity), pH, Glucose, Protein, Bilirubin, Urobilinogen, ketone, Blood, Nitrite, Leukocytes.

-TSH (thyroid function test, once at baseline for abnormal findings)

-Electrocardiogram (once at baseline).

**(4) Body measurements**

-Assessments at baseline and 6 months.

-Blood pressure, height, weight, waist circumference, BMI (Body Mass Index).

**9. Outcomes assessed in the analysis**

Effectiveness outcomes include:

-Changes from baseline to 6 months in fasting blood glucose, HbA1c, TG, T-Cholesterol, HDL-Cholesterol, LDL-Cholesterol, BUN, Creatinine, AST, and ALT.

-Changes from baseline to 6 months in blood pressure, waist circumference, and BMI.

-Changes from baseline to 6 months in dietary, lifestyle (smoking, exercise), and medication use.

**10. Statistical analysis**

**-** Sample size: The sample size was determined using standard statistical criteria (alpha=.05, power=.80) based on the clinical outcome, which was systolic blood pressure between telemonitoring and control in the previous study (McManus et al. 2010). The minimum sample size calculated was 165 patients per group.

-All analyses were performed using IBM SPSS 24 at a significance level of .05. 540 patients were enrolled in the TM intervention group. The Full Analysis Set (FAS) included 1004 subjects (502 in the TM intervention group and 502 in the UC control group) excluding any patients for whom it was documented that they had provided no 6 months follow up data. The FAS was the primary set used for effectiveness analyses. Effectiveness data were analyzed using descriptive statistics. The effectiveness outcomes of the two groups were compared using the chi-square test for categorical variables, and t-test.

-A post hoc analysis was conducted using individual patient data to utilize it as a source of effectiveness data for this economic evaluation study. Observed data at each time point, with no imputation for missing values, were employed as input values for the cardiovascular disease (CVD) risk prediction model. The input variables encompassed demographic characteristics and clinical outcomes associated with CVD risk factors.

**11. Data quality assurance**

**(1) Source data and records**

All data entered in the case report form (CRF) were supported by source data in the patients’ records. The Investigator allowed to authorize co-Investigators and care coordinators to enter study data into the CRF.

**(2) Monitoring**

On-site monitoring was conducted periodically to review all trial-related source data/records, verify the adherence to the protocol and the completeness and accuracy of all CRF entries compared to source data. Monitors were granted direct access to the source data to examine and verify any records and reports that were important to the evaluation of the trial. The monitor issued queries based on abnormal data, which were addressed by investigators. The investigators cooperated with monitors to ensure that any discrepancies identified were resolved.

**Reference**

McManus RJ, Mant J, Bray EP, Holder R, Jones MI, Greenfield S, et al. Telemonitoring and self-management in the control of hypertension (TASMINH2): a randomised controlled trial. Lancet 2010;376(9736):163-72 [doi: 10.1016/S0140-6736(10)60964-6] [Medline: 20619448]
