# Supplementary material for: Information and Communications Technology–Based Monitoring Service for Tailored Chronic Disease Management in Primary Care: Cost-Effectiveness Analysis Based on ICT-CM Trial Results
Source: J Med Internet Res. 2024 Oct 11;26:e51239. doi: 10.2196/51239 (PMC11512140; doi:10.2196/51239)

Table S1^a^. Model inputs for subgroups of patients with hypertension and diabetes.

| Parameters | Patients with hypertension | | Patients with diabetes | | Sources |
| --- | --- | --- | --- | --- | --- |
| **Probability of CVD^b^ events (1-year risk)** | | | | | ICT-CM^c^ trial and CVD Risk Prediction Models |
| **ICT-based tailored management** | Framingham model | KH-CVD^d^ model | Framingham model | UKPDS^e^ model |  |
| 60–64 years | 0.91% | 0.54% | 1.14% | 0.81% |  |
| 65–69 years | 0.91% | 0.54% | 1.14% | 0.81% |  |
| 70–74 years | 1.34% | 0.79% | 1.65% | 1.17% |  |
| 75–79 years | 1.34% | 0.79% | 1.65% | 1.17% |  |
| ≥80 years | 1.82% | 1.08% | 2.24% | 1.59% |  |
| **Usual care** |  |  |  |  |  |
| 60–64 years | 1.37% | 0.84% | 1.51% | 1.10% |  |
| 65–69 years | 1.37% | 0.84% | 1.51% | 1.10% |  |
| 70–74 years | 1.91% | 1.17% | 2.11% | 1.53% |  |
| 75–79 years | 1.91% | 1.17% | 2.11% | 1.53% |  |
| ≥80 years | 2.59% | 1.58% | 2.87% | 2.08% |  |
| **Utility weights, mean (SE)** | | | | | 2007–2019 KNHANES^f^ database |
| **CVD** | | | | |  |
| 60–69 years | 0.860 (0.007) | | 0.850 (0.010) | |  |
| 70–79 years | 0.793 (0.008) | | 0.790 (0.013) | |  |
| ≥80 years | 0.743 (0.016) | | 0.747 (0.032) | |  |
| **Well** | | | | |  |
| 60–69 years | 0.912 (0.002) | | 0.912 (0.003) | |  |
| 70–79 years | 0.856 (0.003) | | 0.857 (0.005) | |  |
| ≥80 years | 0.804 (0.006) | | 0.785 (0.016) | |  |
| **Intervention costs (US $^g^)** | | | | | ICT-CM trial |
| **Bluetooth-enabled devices costs per patient, one-off** | Sphygmomanometer | | Glucometer | |  |
|  | 54.5 | | 34.1 | |  |
| **Equipment costs** | | | | |  |
| Initial development costs of application and operation web per five years | 757,575.8 | | 757,575.8 | |  |
| Annual network use costs | 227,272.7 | | 227,272.7 | |  |
| Annual system update costs | 145,454.5 | | 145,454.5 | |  |
| Total costs per patient, annuitized based on lifespan of five years | 2.3 | | 2.4 | |  |
| **Running costs per patient^h^** | 141.8 | | 141.8 | |  |
| **Treatment costs of health states (US $)** | | | | | Analysis using national health insurance claims data by NHIS^i^ |
| **Acute phase CVD (first year costs after event)** | | | | |  |
| 60–64 years | 2650.1 | | 3321.1 | |  |
| ≥65 years | 3523.1 | | 4329.2 | |  |
| **Chronic phase CVD (annual costs in subsequent years)** | | | | |  |
| 60–64 years | 1564.0 | | 2010.3 | |  |
| ≥65 years | 2571.0 | | 2963.8 | |  |
| **Well (annual costs)** | | | | |  |
| 60–64 years | 890.8 | | 1180.7 | |  |
| ≥65 years | 1511.3 | | 1825.9 | |  |

^a^Table S1 listed only inputs unique to each subgroup, differing from the values applied to the total cohort. In other words, the parameters such as analysis setting, probability of death due to CVD, probability of death due to other causes, and age-related relative risk of CVD, not listed in Table S1, have the same inputs as those applied to the total patients included in Table 2.

^b^CVD: cardiovascular diseases.

^c^ICT-CM: Information and communications technology (ICT)-based tailored chronic disease management.

^d^KH-CVD: Korean Hypertension cardiovascular.

^e^UKPDS: United Kingdom prospective diabetes study.

^f^KNHANES: Korea National Health and Nutrition Examination Survey.

^g^All costs are expressed in 2023 US $ using an exchange rate of US $1 to 1320 KRW.

^h^Through the ICT-TM trial, the annual running cost was calculated by dividing the care coordinator's labor cost by the number of managed patients (approximately 250 per year).

^i^NHIS: National Health Insurance Service.

Figure S1. Tornado diagram for TM versus UC. CVD: cardiovascular diseases; TM: ICT-based tailored management; UC: usual care.


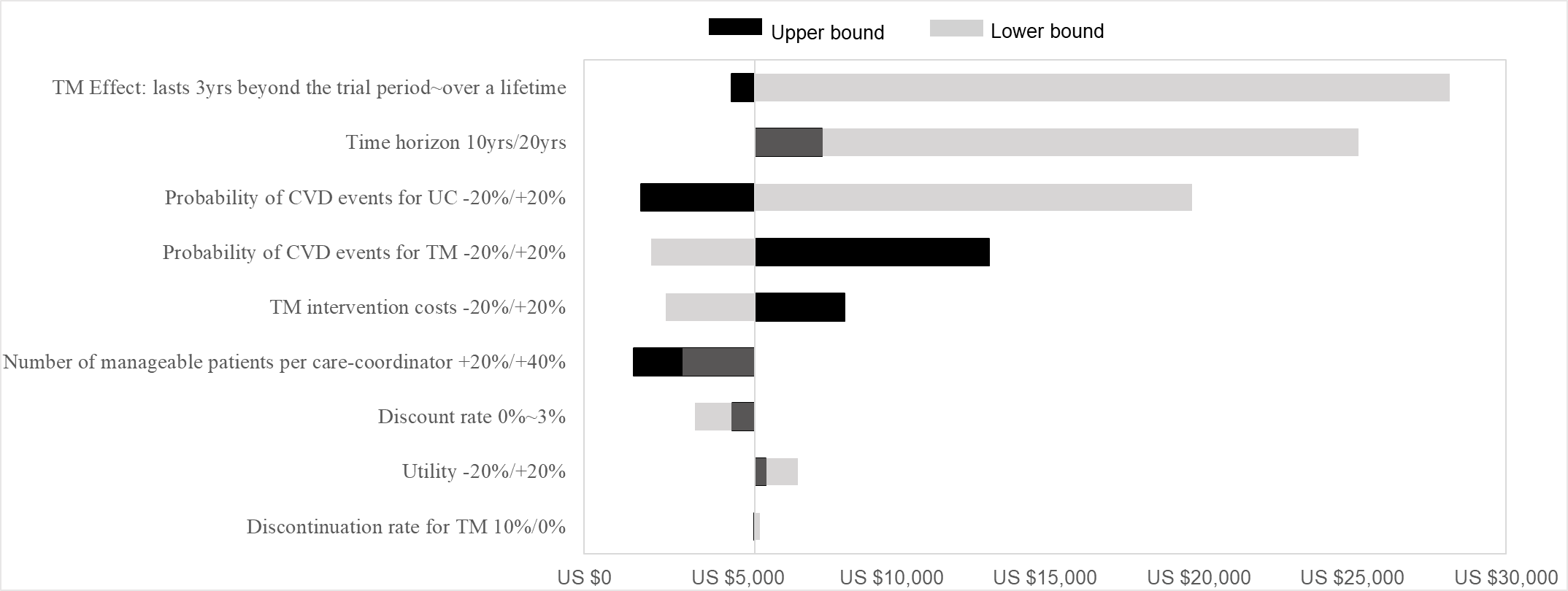


**Figure S2**. Results of probabilistic sensitivity analysis for subgroups of patients with hypertension and diabetes. (A) Cost-effectiveness plane for patients with hypertension. (B) Cost-effectiveness acceptability curve for patients with hypertension. (C) Cost-effectiveness plane for patients with diabetes. (D) Cost-effectiveness acceptability curve for patients with diabetes. Dotted line on graph indicates an incremental cost-effectiveness ratio threshold. ICER: incremental cost-effectiveness ratio; ICT: information and communications technology; QALY: quality-adjusted life-year.


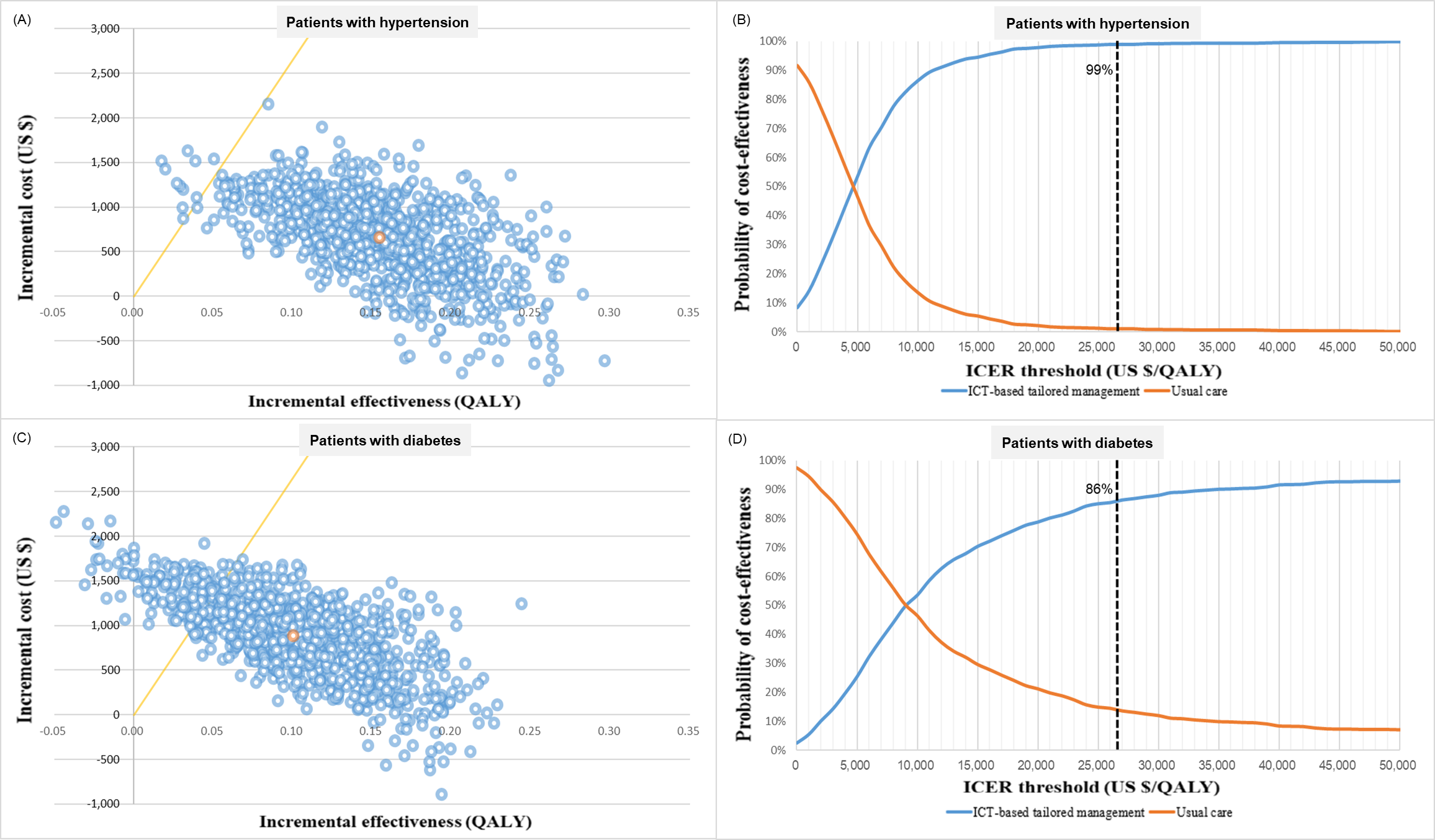

Supplement: Multimedia Appendix 2 [file jmir_v26i1e51239_app2.docx]
